# Detection of *Listeria monocytogenes* in foods with a textile organic electrochemical transistor biosensor

**DOI:** 10.1007/s00253-023-12543-y

**Published:** 2023-05-05

**Authors:** Priya Vizzini, Elena Beltrame, Nicola Coppedè, Filippo Vurro, Francesco Andreatta, Emanuela Torelli, Marisa Manzano

**Affiliations:** 1 Department of Agriculture Food Environmental and Animal Sciences, University of Udine, 33100 Udine, Italy; 2 Institute of Materials for Electronics and Magnetism IMEM, CNR Parco Area delle Scienze, 43124 Parma, Italy; 3 Polytechnic Department of Engineering and Architecture, University of Udine, 33100 Udine, Italy; 4 Interdisciplinary Computing and Complex BioSystems (ICOS), Centre for Synthetic Biology and Bioeconomy (CSBB), Devonshire Building, Newcastle University, Newcastle upon Tyne, NE1 7RX UK

**Keywords:** OECT biosensor, *Listeria monocytogenes*, AFM, Ready-to-eat food, DNA probe

## Abstract

**Abstract:**

Foods contaminated by pathogens are responsible for foodborne diseases which have socioeconomic impacts. Many approaches have been extensively investigated to obtain specific and sensitive methods to detect pathogens in food, but they are often not easy to perform and require trained personnel. This work aims to propose a textile organic electrochemical transistor-based (OECT) biosensor to detect *L. monocytogenes* in food samples. The analyses were performed with culture-based methods, *Listeria* Precis™ method, PCR, and our textile OECT biosensor which used poly(3,4-ethylenedioxythiophene) (PEDOT):polystyrene sulfonate (PSS) (PEDOT:PSS) for doping the organic channel. Atomic force microscopy (AFM) was used to obtain topographic maps of the gold gate. The electrochemical activity on gate electrodes was measured and related to the concentration of DNA extracted from samples and hybridized to the specific capture probe immobilized onto the gold surface of the gate. This assay reached a limit of detection of 1.05 ng/μL, corresponding to 0.56 pM of *L. monocytogenes* ATCC 7644, and allowed the specific and rapid detection of *L. monocytogenes* in the analyzed samples.

**Keypoints:**

• *Textile organic electrochemical transistors functionalized with a specific DNA probe*

• *AFM topographic and surface potential maps of a functionalized gold gate surface*

• *Comparison between the Listeria monocytogenes Precis™ method and an OECT biosensor*

**Supplementary Information:**

The online version contains supplementary material available at 10.1007/s00253-023-12543-y.

## Introduction

*Listeria monocytogenes* is a pathogen responsible for listeriosis, a disease which causes fatal cases due to ingestion of contaminated food, especially ready-to-eat (RTE) products (smoked salmon, dry-cured ham), dairy products, meat, fish, and vegetables (EFSA 2015a, b; EFSA 2016; Katzav et al. 2006). The current ISO 11290 detection method requires long analysis time related to the enrichment step, the isolation on differential media, and the biochemical tests. The rapid detection of *L. monocytogenes* is an important challenge for food industry to reduce economic losses or recalls of products (Jemmi and Stephan 2006). Molecular methods like PCR and qPCR reduce the analysis time: nevertheless, new approaches involving the construction of biosensors are required to shorten the assay time and to improve routine food analysis. In particular, the DNA-based biosensors have the advantage of being highly specific when compared to immunosensors and harmless in comparison with viable pathogens cells, which represent a great risk for the operator and require the implementation of procedures for hazardous waste handling and disposal (Manzano et al. 2015; Vizzini et al. 2019; Wu et al. 2019).

Organic electrochemical transistors (OECTs) are a combination of a sensor and an amplifier consisting of a channel made of an organic p-type semiconductor with source and drain contacts and a gate electrode immersed into an electrolytic solution (Battista et al. 2017; Coppedè et al. 2014a; He et al. 2012; Peng et al. 2018; Tao et al. 2017). OECTs are sensitive, potentially low cost, and biocompatible as operating at low voltage and can be easily miniaturized or fabricated on flexible substrates (Coppedè et al. 2014b; Lin et al. 2011).

OECTs are electrochemical transistors, with a three-electrode architecture, realized with source and drain electrodes placed at opposite sides of an organic layer forming the channel and the third electrode (gate) connected with the channel by an electrolyte solution (Paudel et al. 2021). OECTs are used as biosensors in liquid environment due to the de-doping properties of their organic channel, usually based on poly(3,4-ethylenedioxythiophene) polystyrene sulfonate (PEDOT:PSS) p-type doped conductive polymer, as material of election. In fact, in correspondence with the application of a potential to the gate, the ions in the electrolyte are forced to enter the channel, causing a de-doping in the organic material and a variation in its conductivity that is proportional to the concentration of ions injected from the electrolyte (Rivnay et al. 2018). In such a way, the electrochemical transistor can be used to measure the ion concentration in the electrolyte. To improve the selectivity properties of OECT, specific functionalization needs to be added to the device geometry.

He et al. (2012) built an OECT biosensor using antibodies against *Escherichia coli* (attached to the surface of the PEDOT:PSS layer) for its detection, while Hai et al. (2018) built an OECT to detect human influenza A virus by the utilization of 2,6-sialyllactose protein to functionalize the channel. As reported by Sophocleous et al. (2021), OECT can be considered a biosensing platform useful for various applications, including specific microbial detection.

Lin et al. (2011) described an OECT biosensor functionalized with single-stranded (ss) DNA probes immobilized on the surface of Au gate electrodes to detect complementary DNA targets reaching a sensitivity of 1 nM. Liang et al. (2019) described an OECT aptasensor for ATP evaluation which reached a sensitivity of 10 pM, while Peruzzi et al. (2021) developed an OECT with a graphene gate electrode and the addition of nanoparticles to detect thrombin reaching a limit of detection (LOD) of 5 pM.

He et al. (2012) utilized an organic electrochemical transistor to detect *Escherichia coli* in KCl electrolyte, while here a ssDNA probe designed for the specific detection of *L. monocytogenes* in food samples was used. Our OECT is a label-free genosensor which can be easily functionalized with other DNA probes, specific for different food pathogens, and used for routine detection in food industries.

**Fig. 1:**
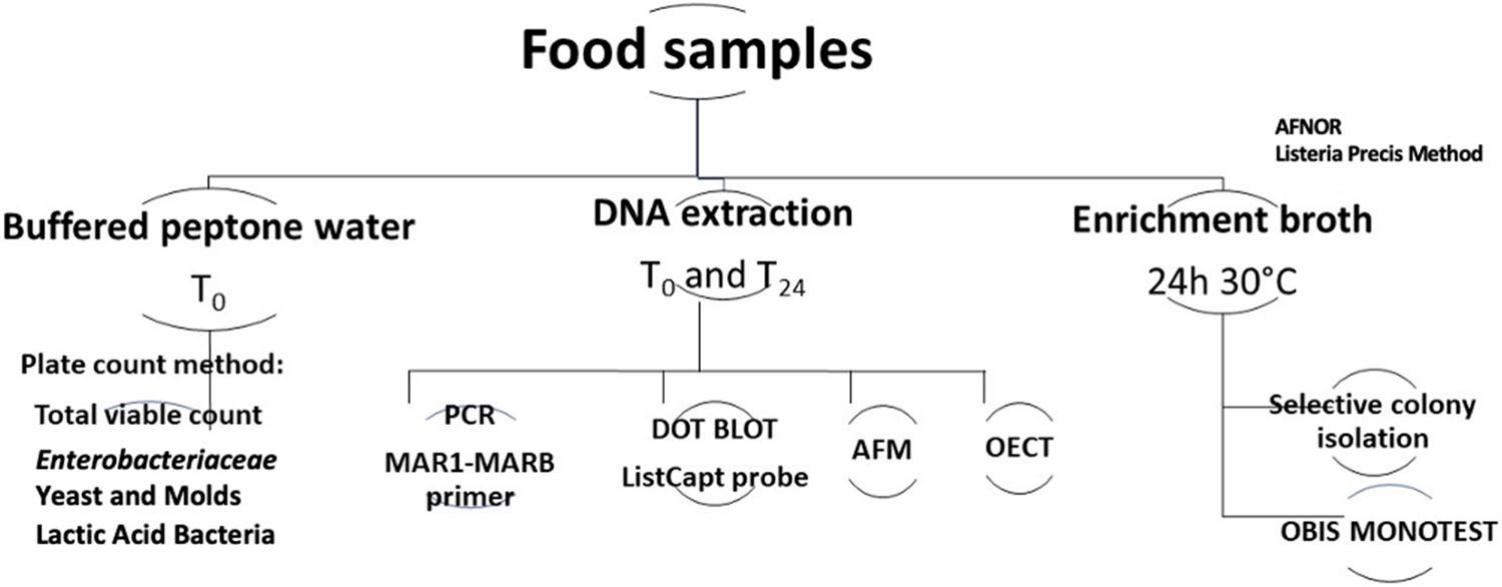
Workflow of the experiment

The aim of this work was to evaluate a textile OECT as a simple DNA-based sensor to rapidly detect *L. monocytogenes* in ready-to-eat foods (smoked salmon, fresh ham, and cured ham): in detail, the results derived from our biosensor were compared with *Listeria* Precis™ method and qPCR. The textile OECT biosensor was built with a specific bioreceptor DNA probe and a PEDOT:PSS p-type doped polymer for the textile-based channel and could represent a good alternative for rapid and low-cost diagnostics in various applications (Fig. 1).

## Materials and methods

### Materials and reagents

Phosphate buffer saline (PBS) 1X (pH 7.4) and bovine serum albumin (BSA) were purchased from Sigma-Aldrich (Milan, Italy). DNA primers and probe were synthesized by MWG-Biotech (Ebersberg, Germany). Reagents for PCR were purchased from Applied Biosystems (Milan, Italy). The nylon membrane (Zeta-Probe GT; Bio-Rad, Milan, Italy) and Dig Easy Hyb buffer (Roche Diagnostics, Monza, Italy) were used in the dot blot tests. For the biosensor construction, PEDOT:PSS (Clevios™ PH500, Starck GmbH, Cologne, Germany), ethylene glycol (Sigma-Aldrich, Milan, Italy), dodecyl benzene sulfonic acid (DBSA) (Sigma-Aldrich, Milan, Italy), and PBS 1X were used. All media used for the microbiological analysis were purchased from Oxoid (Milan, Italy). BioNano gold-coated borosilicate coverslips of 15 mm × 13 mm with gold thickness of 50 nm on a glass substrate of 15 mm × 15 mm × 0.15 mm (PHASIS, Geneva, Switzerland) were used to build the textile OECT biosensor.

### Microorganisms and food samples

The reference microorganisms listed in Table 1 were used to test the specificity of primers and probe, thus optimizing the biosensor working protocol. A total of eight samples were tested, four sliced smoked samples (ready-to-eat) purchased from local supermarkets (packages of 100 g) and four hams from ham factories in Friuli Venezia Giulia Region (Italy).

**Table 1:** List of the microorganisms used

1	Positive controls	*Listeria monocytogenes* 1/2c	^*^ATCC 7644
2		*Listeria monocytogenes* 1/2a	^*#*^DSM 112143
3		*Listeria monocytogenes* 1/2b	^#^DSM 19094
4		*Listeria monocytogenes* 4b	^*#*^DSM 15675
5	Negative controls	*Listeria innocua*	^*#*^DSM 20649
6		*Listeria ivanovii subsp. iondoniensis*	^*#*^DSM 12491
7		*Salmonella enterica*	^*#*^DSM 9145
8		*Escherichia coli*	^*#*^DSM 1103
9		*Bacillus cereus*	^*#*^DSM 2301
10		*Campylobacter jejuni*	^*#*^DSM 49943
11		*Lactiplantibacillus plantarum*	^*^ATCC BAA-793
12		*Lacticaseibacillus rhamnosus*	^*^ATCC 53103
13		*Lacticaseibacillus paracasei*	^*#*^DSM 5622
14		*Levilactobacillus brevis*	^*#*^DSM 20054

^*^ATCC American Type Culture Collection (Manassas, VA, USA)

^#^*DSM* Deutsche Sammlung von Mikroorganismen und Zellkulturen GmbH (Braunschweig, Germany)

Two samples of smoked salmon were analyzed at purchasing (SS1_p_, SS2_p_) and two after four weeks at + 4 °C (SS1_4w_, SS2_4w_). Two samples of fresh ham (RH1, RH2) (before seasoning) and two of dry-cured ham (CH1, CH2) (at 24 months seasoning) were used for the analyses too.

### Food sample analysis: plate count and Listeria Precis™ methods

Samples were subjected to (i) traditional plate count method for the determination of the total viable count, *Enterobacteriaceae*, lactic acid bacteria, yeasts, and molds; (ii) the *Listeria* Precis™ method (NF Validation EN ISO 2003) for the presence of *L. monocytogenes*; and (iii) OECT analysis (Fig. 1).

#### Plate count method

10 g of each sliced salmon sample (SS) was transferred into a sterile Stomacher bag, added with 40 mL of buffered-peptone water (BPW), mixed for 30 s in a Stomacher (PBI, Milan, Italy), and used for plate count bacterial enumeration and DNA extraction. An area of (10 cm × 10 cm) 100 cm^2^ for each ham sample was sampled using sterile gauzes of (3.5 cm × 7 cm) 24.5 cm^2^ hydrated with 3 mL of BPW (1 g/L peptone and 8 g/L NaCl) to recover the microbial content of the surfaces. The hydrated gauzes used for the surface sampling were suspended in 10 mL of BPW, shaken with a vortex for 10 s, and used for plate count bacterial enumeration and DNA extraction. Total viable count on Tryptone Soya Agar (TSA) (30 °C for 48 h), lactic acid bacteria on de Man Rogosa Sharpe (MRS) (30 °C for 48 h) in AnaeroJar 2.5 L (Oxoid) with a candle for microaerophilic conditions, *Enterobacteriaceae* on a double layer of Violet Red Bile Glucose (VRBG) agar (37 °C for 24 h), yeasts and molds on malt extract agar added with 10 μg/mL of tetracycline (Sigma-Aldrich, Milan, Italy) (30 °C for 48 h) were evaluated.

## *Listeria*Precis ™ method

25 g of smoked salmon from each sample was transferred into a sterile Stomacher bag, added with 225 mL of ONE Broth-*Listeria*, and incubated for 24 h at 30 °C, whereas for fresh ham and cured ham, 3 mL from the BPW dilutions used for microbial enumeration was transferred into 27 mL of ONE Broth-*Listeria* and incubated for 24 h at 30 °C. The selective isolation of *L. monocytogenes* was performed by streaking 10 μL from the enriched broths on Brilliance *Listeria* agar and incubating at 37 °C for 24 h. Presumptive *L. monocytogenes* blue/green colonies with haloes were confirmed using the O.B.I.S. Mono test (Oxoid). The enrichment broths were used for DNA extractions at 24 h incubation.

### Primers and DNA probe

*Listeria* spp. *iap* gene sequences were retrieved from GenBank and aligned using the “multiple sequence alignment with hierarchical clustering” (Corpet 1988) to design a specific reverse primer (Mar B, 5′-TCA GCT GCT GGA GCT TC-3′, 17 bp, 58% GC, Tm 53 °C) to be coupled to the Mar1 forward primer (19 bp, 36.8% GC, Tm 46.9 °C) (Manzano et al. 1997) for the specific detection of *L. monocytogenes* in food samples using the end point PCR technique.

The primer set, expected to produce an amplicon of 160 bp, was tested with IDT OligoAnalyzer 3.1 (http://eu.idtdna.com/calc/analyzer) and AmplifX 1.7.0 (Jullien 2013) before their utilization in PCR assays (MWG-Biotech, Ebersberg, Germany). Moreover, a DNA probe specific for *L. monocytogenes* (List Capt) 5′-TAA AAA TAC CAA TAC TAA TAC AAA CTC CAA TAC G-3′ (Fontanot 2014) was tested in silico using BLASTn (Altschul et al. 1990) and IDT OligoAnalyzer 3.1 (http://eu.idtdna.com/calc/analyzer). The probe synthetized by MWG-Biotech (Ebersberg, Germany) with digoxigenin at 5′ end (List Capt-Dig) was tested in vitro by dot blot technique. A sequence (MWG-Biotech, Ebersberg, Germany) complementary to the selected DNA probe was used as a positive control for the hybridization protocols. Both Mar1-MarB primers and the List Capt probe were tested on the DNAs extracted from the bacteria listed in Table 1. After dot blot test, the List Capt probe was labelled with a thiol-C6 (List Capt-SH) at 5′ end to allow the immobilization on the gold gate used for the textile OECT biosensor construction.

### DNA extraction

The DNA of the reference strains reported in Table 1 was extracted according to Manzano et al. (2003). DNA from samples was extracted as follows: 2 mL homogenates were collected from the Stomacher bag of each sample (from buffered peptone water (BPW) and ONE Broth-*Listeria*) and centrifuged for 10 min at 13,000 rpm. After discarding the supernatant, the pellet was suspended in 300 μL of breaking buffer (2% Triton X-100, 1% SDS, 100 mM NaCl, 10 mM Tris–HCl, 1 mM EDTA pH 8, Sigma-Aldrich) and subjected to the protocol described previously (Manzano et al. 2003). Extracted DNAs were quantified with a spectrophotometer (NanoDrop 2000C, Thermo Fisher, Milan, Italy), standardized at 100 ng/μL, and stored at − 20 °C until analysis by PCR and dot blot.

### PCR protocol

PCR was carried out using a mixture containing 5 μL AmpliTaq® buffer 10x (Applied Biosystems), 1 μL MgCl_2_ 25 mM (Applied Biosystems), 1 μL PCR Nucleotide Mix 10 mM each (Applied Biosystems), 1 μL of each primer (Mar1 and MarB at 10 mM), and 0.25 μL AmpliTaq® DNA Polymerase 5 units/μL (Applied Biosystems) in a final volume of 50 μL. The conditions applied were as follows: 95 °C denaturation for 5 min, followed by 30 cycles 95 °C for 1 min, annealing at 48 °C for 45 s, extension at 72 °C for 30 s, and final extension at 72 °C for 7 min.

### Dot blot test

The List Capt-Dig probe was used in the dot blot tests for specificity at 100 ng/μL. 1 μL of each DNA extracted from the reference strains listed in Table 1 (at 100 ng/μL) was spotted onto a positively charged nylon membrane (Zeta-Probe GT) after 10 min denaturation at 95 °C and cross-linked by exposure to UV light (254 nm) for 10 min. The membrane hybridization was carried out overnight at 40 °C in Dig Easy Hyb buffer (Roche Diagnostics, Monza, Italy) according to Cecchini et al. (2012) protocol.

### Textile organic electrochemical transistor (OECT) biosensor construction

The textile organic electrochemical transistor (OECT) working principle is based on the de-doping effect on the polymeric channel performed upon the application of a positive potential at the gate electrode that is applied through the ionic species in the fluid sample. The OECT response, calculated as the variation of *I*_ds_ current upon the application of gate potential, depends on the potential drops occurring at the gate/electrolyte and electrolyte/polymer interfaces. The application of gate potential forces cations to move toward the polymer surface at the channel and de-dope poly(3,4-ethylenedioxythiophene) (PEDOT):polystyrene sulfonate (PSS) (PEDOT:PSS). The sensing mechanism induces a reduction of the holes along the PEDOT:PSS channel, due to de-doping of cations, generating a reduction in the drain–source current (*I*_ds_) module proportional to the concentration of the analyte. The DNA sample, interacting at the gate interface, will modify the capacitance of the interface, varying the applied potential on the channel. Hence, a variation due to the presence of a DNA sample can be detected by OECT by measuring the related variation of the *I*_ds_ response.

The textile OECT biosensor used was built with a channel constituted by a polypropylene textile fiber functionalized with a conductive polymer, the aqueous PEDOT doped with PSS (PEDOT:PSS, Clevios PH500, Starck GmbH, Munich, Germany) (Tarabella et al. 2012). The polymer was added with ethylene glycol (10% v/v) and dodecyl-benzene-sulfonic acid (2% v/v) to increase conductivity and the adhesion to the fiber. The final solution obtained was used for the functionalization of the textile fiber by the drop casting technique, and the fibers were then baked at 130 °C for 90 min. Before functionalization, each thread was cleaned by means of plasma oxygen cleaner treatment (Femto, Diener electronic, Ebhausen/Germany) to increase its wettability and facilitate the adhesion of the aqueous conductive polymer solution. The channel was 14 mm long and 2 mm width. The channel surface has been estimated to be 0.28 cm^2^. The gate area corresponds to the surface of the metal gate electrode in direct contact with the electrolyte. The surface of the metal electrode is 2.4 cm^2^.

The channel area corresponds to the fiber surface covered with the organic semiconductor in direct contact with the electrolyte. All the fiber immersed in the electrolyte solution is working as a channel. The contact at the fiber edges at the external of the cell is made by metal wires and isolated with an insulating polymer.

**Fig. 2:**
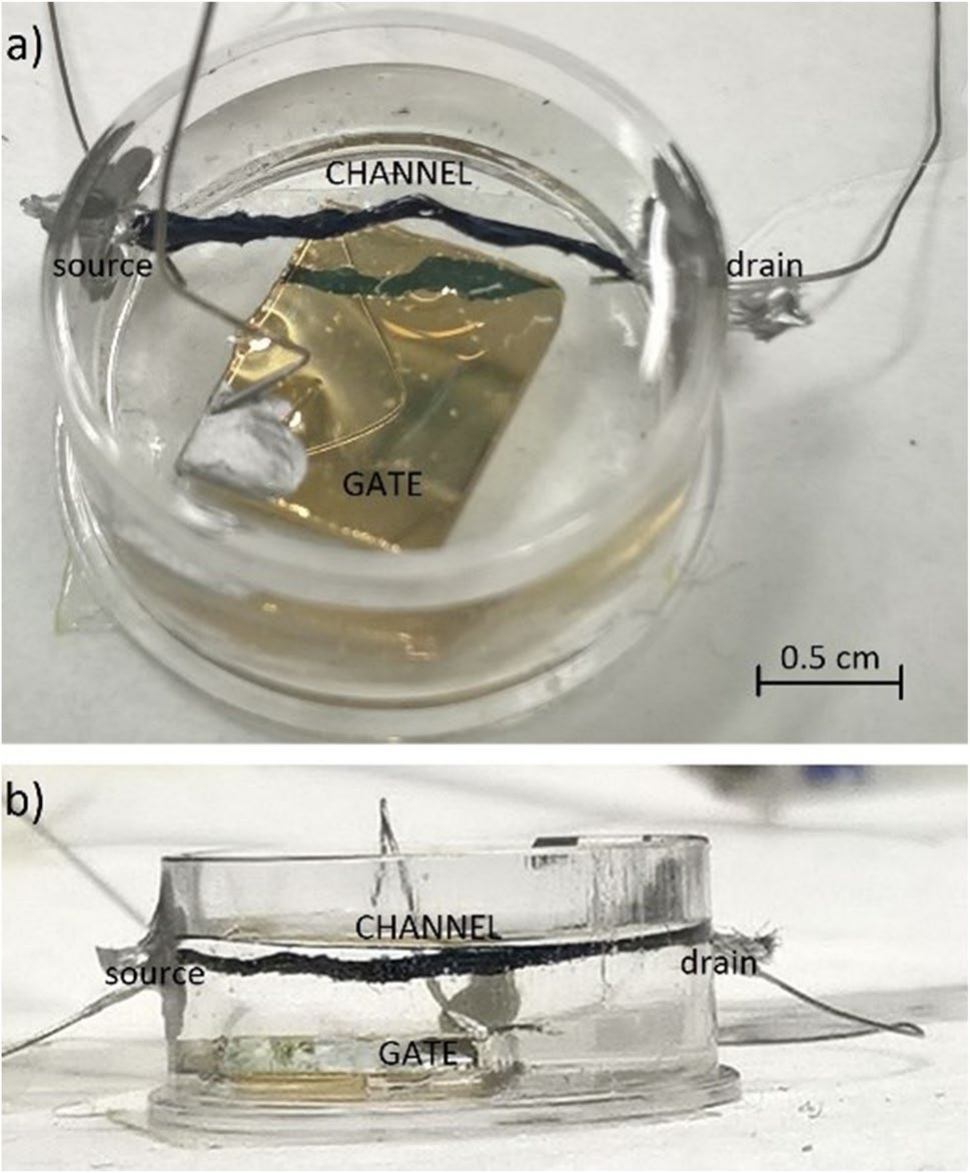
Textile organic electrochemical transistor (OECT) biosensor used for the analyses of the ready-to-eat food samples. The metal electrode of 2.4 cm^2^ is covered with 3 mL solution of the organic semiconductor in contact with the electrolyte; the channel is 14 mm long and 2 mm wide with a surface of about 0.28 cm^2^. The gold gate is functionalized with 10 ng/μL List Capt-SH probe

The transistor device was completed with the immobilization of the capture probe (List Capt-SH) on the gold gate after activation by de-protecting the thiol group following the manufacturer’s protocol (Fig. 2).

The List Capt-SH probe was used at 10 ng/μL in PBS 1X and kept for 1 h at room temperature (RT) before washing to remove the not bound probe. Then, the surface was blocked with MCH 1 mM in PBS for 1 h at RT and washed. 10 μL of each DNA dilution (in PBS 1X) from pure cultures of *L. monocytogenes* ATCC 7644 (0.1, 1, and 10 ng/μL), *L. innocua* DSM 20,649 (100 ng/μL), and from food samples was spotted onto the functionalized gold gate after 10 min denaturation at 95 °C and incubated 1 h at 40 °C. The gold gate electrodes were rinsed twice with 500 μL sterile distilled water (S-ddwater), to remove the excess DNA, air-dried under a sterile laminar flow cabinet and kept in a dryer jar until the measurements conducted at room temperature (RT).

### AFM images of Au electrode before and after functionalization

An atomic force microscope (AFM) was employed in this work for the acquisition of topographic maps of the gold substrates employed for the construction of the biosensors before (bare gold) and after functionalization with the DNA probe. The AFM employed in this work is equipped with the scanning Kelvin probe force microscopy technique (SKPFM), which enables the acquisition of surface potential maps of the same regions of the substrates where topographic images were recorded. The measurements were carried out using a Nanoscope III multimode atomic force microscope (AFM) equipped with an Extender TM electronic module enabling the simultaneous acquisition of topographic and surface potential maps. The topographic maps were recorded in tapping mode while the surface potential maps were obtained in lift mode by means of the SKPFM technique. All measurements were performed using n^+^ silicon tips coated with PtIr_5_ at room temperature with a relative humidity of 40–65%.

The scan frequency was 0.1 Hz (256 × 256 lines), and the scan height in lift mode was 100 nm. Modifications of topography and surface potential were evaluated for the following samples: bare gold substrate, gold substrate functionalized with the List Capt-SH probe, and functionalized gold substrate after hybridization with the DNA of *L. monocytogenes.* The images were processed by the software Nanoscope III—Digital Instruments—version 5.30r3.sr3.

### Electrochemical measurement

Between the physical quantities that an OECT device measures the most important are the drain-source current and the gate-source current, respectively, passing through the channel and the liquid of the system. These currents are originated by two applied voltages, the drain-source voltage (*V*_ds_) applied on the channel and the gate-source voltage (*V*_gs_) applied on the gate, which lead to the generation of two interfaces in the OECT device: the gate/electrolyte and the channel (PEDOT:PSS)/electrolyte interfaces. The electrolyte is a PBS solution. The OECT measurements have been performed with a high-precision two-channel electrometer Agilent B2902A.

Each interface is characterized by an electric double layer (EDL) to generate the gate and channel capacitance (*C*_g_ and *C*_c_, respectively) that are connected in series: the gate voltage (*V*_gs_) is distributed at the two interfaces (Bernards et al. 2008; Cicoira et al. 2010). In this system, the p-type doped nature of the semiconductor PEDOT (oxidized from the electrochemistry point of view) leads to mobile hole that drift along the polymer generating a hole current (*I*_ds0_) which flows in the channel when a drain voltage (*V*_ds_ =  − 0.05 V) is applied. These holes are balanced by the negative charge of the PSS sulfonate group (Elschner et al. 2018) until the application of a positive gate bias (gate voltage from 0.2 V to 1 V, with 0.2 step voltage) which leads to the injection of the cations (M^+^) from the electrolyte (PBS) into the PEDOT:PSS channel causing its de-doping, according to equation (Nilsson et al. 2005).

1 $${\mathrm{PEDOT}}^{+}:{\mathrm{PSS}}^{-}+{\mathrm{M}}^{+}+{\mathrm{e}}^{-}\to {\mathrm{PEDOT}}^{0}+{\mathrm{M}}^{+}:{\mathrm{PSS}}^{-}$$

Moreover, the “de-doping process” (Bernards et al. 2008), according to the reduction of the oxidized PEDOT^+^ to PEDOT^0^ and the decrease of the number of holes in the channel, leads to drop in the drain current (*I*_ds_). At least, the whole process is reversible, so when gate-source voltage is switched off (*V*_gs_ = 0 V), cations diffuse from the channel to the electrolyte, increasing the number of conducting holes, and consequently, reduced PEDOT^0^ returns to the oxidized state and drain-source current to the initial value (*I*_ds0_).

The behavior of the resulting drain-source current from these processes was monitored over time and the sensor response parameter *R* was expressed by

2 $$R=\left|{I}_{\mathrm{dS}}-{I}_{\mathrm{dS}0}\right|/{I}_{\mathrm{dS}0}$$

## Results

### Plate count bacterial enumeration and Listeria Precis™ method

The microbiological data related to the sliced salmon samples and fresh and cured ham samples are reported in Table 2, together with positive and negative results from the *Listeria* Precis™ method, PCR, and OECT*.*

**Table 2:** Results of the plate count data of samples expressed as colony-forming unit (CFU)/g for sliced smoked salmon and CFU/cm^2^ for fresh ham and dry-cured ham

Samples	Total viable count	Lactic acid bacteria	*Enterobacteriaceae*	Yeasts	Molds	*Listeria* Precis™ method	PCR	OECT delta response (∆*R*)
a. Sliced smoked salmon								
SS1_p_	1.7 × 10^6^	8.7 × 10^4^	< 5^*^	< 25^*^	< 25^*^	Negative	Negative	0.0038
SS2_p_	2.5 × 10^4^	< 2.5 × 10^4*^	< 5^*^	3.7 × 10	< 25^*^	Negative	Negative	0.0025
SS1_4w_	1.8 × 10^8^	5.3 × 10^7^	< 5^*^	1.5 × 10^5^	< 25^*^	Negative	Negative	0.0046
SS2_4w_	1.7 × 10^6^	< 2.5 × 10^4*^	5.2 × 10^1^	2.6 × 10^2^	< 25^*^	Positive	Positive	0.1540
b. Raw ham and dry-cured ham								
RH1	9.1 × 10^3^	1.9 × 10^3^	9.9 × 10^2^	6.6 × 10^2^	< 130	Negative	Negative	0.0039
RH2	2.5 × 10^4^	7.9 × 10^3^	3.3 × 10	7.4 × 10^2^	< 130	Positive	Positive	0.0287
CH1	1.1 × 10^4^	< 130	< 33	1.7 × 10^3^	< 130	Negative	Negative	0.0019
CH2	7.2 × 10^4^	< 130	< 33	1.5 × 10^3^	< 130	Negative	Negative	0.0043

*SS*_*p*_ samples analyzed at purchasing, *SS*_*4w*_ samples analyzed after 4 weeks, *RH* raw ham at *t*0, *CH* dry-cured ham at *t*0

^*^Limit of detection of the method

The data of the total viable count of sliced smoked samples (Table 2 (part a)) ranged from 2.5 × 10^4^ to 1.8 × 10^8^ CFU/g, the lactic acid bacteria ranged from values below the limit of detection of the method to 5.3 × 10^7^ CFU/g, and the *Enterobacteriaceae* were detected only in sample SS2_4w_, with a value of 5.2 × 10^1^ CFU/g. The yeasts ranged from 3.7 × 10 to 1.5 × 10^5^ CFU/g, and sample SS1_p_ was below the limit of detection, while molds were always below the limit of detection. *L. monocytogenes* was only detected in the SS2_4w_ sample.

The data related to the fresh ham (RH samples) and dry-cured ham (CH samples) are reported in Table 2 (part b). Total bacterial count ranged from 9.1 × 10^3^ to 7.2 × 10^4^ CFU/cm^2^. Lactic acid bacteria values were between 1.9 × 10^3^ and 7.9 × 10^3^ CFU/cm^2^ in RH samples and below the limit of detection of the method utilized in CH samples. *Enterobacteriaceae* were detected in RH samples with a value of 9.9 × 10^2^ CFU/cm^2^ while they were not detected in CH samples. Yeasts were found in all samples with counts ranging from 6.6 × 10^2^ to 1.7 × 10^3^. Molds were below 1.3 × 10^2^ CFU/cm^2^. Only sample RH2 resulted positive for *L. monocytogenes*.

### Molecular methods

The couple of primers Mar1-MarB tested in silico and used for an end point PCR on the DNA of the bacteria listed in Table 1 produced the expected amplicons of 160 bp only for the *L. monocytogenes* serotypes 1/2a, 1/2b, and 1/2c (Fig. S1a) and serotype 4b (data not shown).

The absence of the amplification products was observed for *L. innocua* DSM 20649 and *L. ivanovii* DSM 12491 (Fig. S1b) species with a genome with high homology to *L. monocytogenes* genome and used as negative controls, confirming Mar1-MarB primer specificity.

After 24 h incubation in ONE Broth-*Listeria*, only SS2_4w_ and RH2 samples were found positive by PCR using the specific *L. monocytogenes* primers Mar1-MarB (Fig. S1) producing an amplicon of 160 bp; these data were confirmed by the *Listeria* Precis™ method results. The List Capt probe tested by dot blot was specific as demonstrated by the presence of blue spots only for *L. monocytogenes* DNAs (Fig. S2) and not for other bacteria reported in Table 1.

### AFM topographic and surface potential maps

The gold substrates employed for the construction of the biosensors before (bare gold) and after functionalization with the DNA probe were investigated by AFM to obtain topographic maps of the sample surface. The SKPFM technique was used to record surface potential maps of the same regions on which the topographic maps were acquired.

**Fig. 3:**
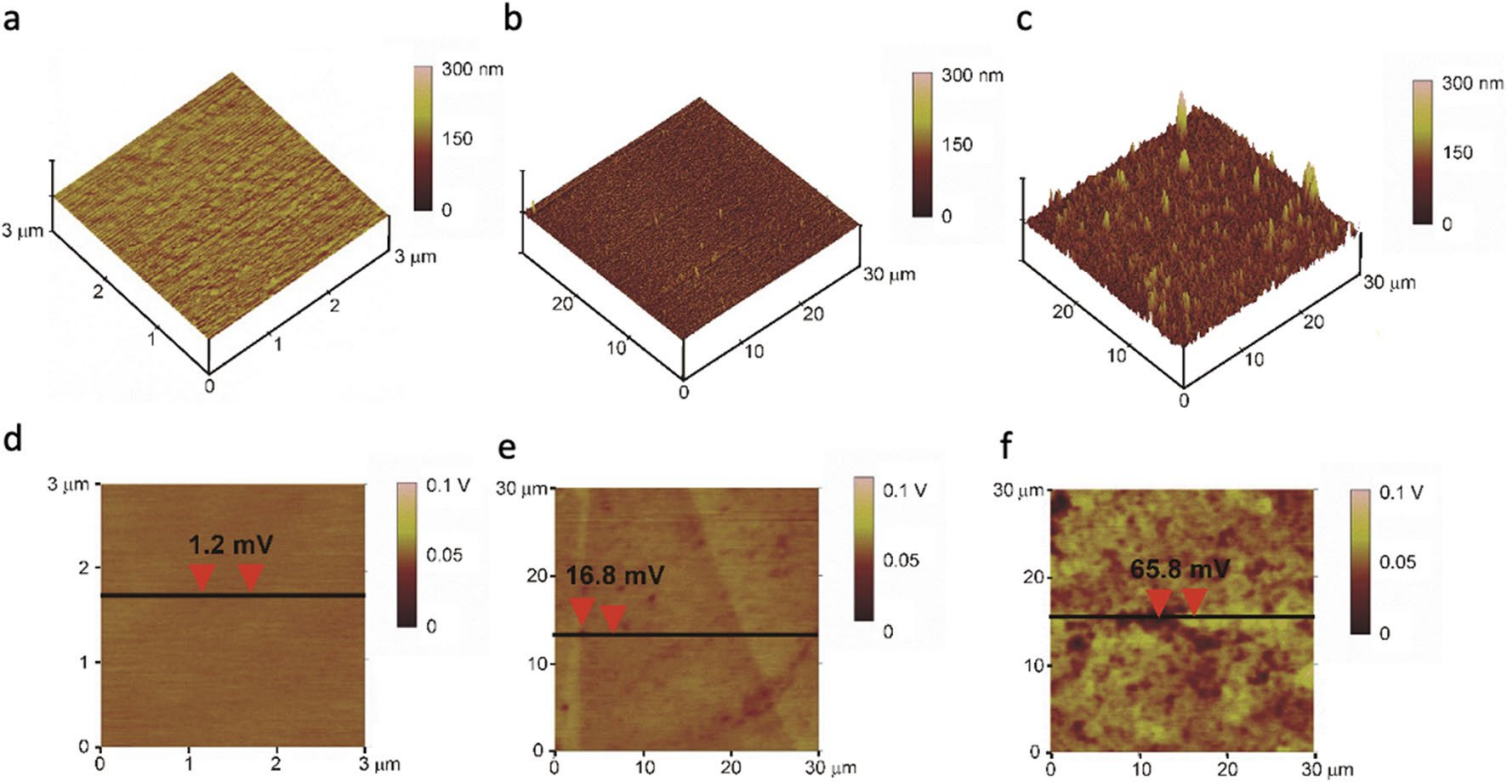
3D topographic maps obtained by AFM of the bare gold substrate (**a**); gold substrate functionalized with List Capt-SH (**b**) and functionalized gold substrate after hybridization with the DNA of *L. monocytogenes* ATCC 7644 (**c**). Surface potential maps obtained by SKPFM technique of bare gold substrate (**d**); gold substrate functionalized with List Capt-SH (**e**); functionalized gold substrate after hybridization with the DNA of *L. monocytogenes* ATCC 7644 (**f**). On the potential maps, the surface potential differences across the regions are indicated by the arrows

Figure 3 displays a 3D topographic map (3 μm × 3 μm) of the gold substrate employed for the construction of the biosensor.

The substrate (bare gold) presents a very uniform and flat surface, with the roughness parameter (*R*_a_) at 0.5 nm indicating a very low roughness (Fig. 3a). Figure 3d (surface potential map of the same region displayed in Fig. 3a) shows very small (1.2 mV) potential differences measured on the surface confirming the uniformity of the substrate also from an electrochemical viewpoint. Figure 3b shows a 3D topographic map (30 μm × 30 μm) of the gold substrate after functionalization with the List Capt-SH probe. The surface retains its uniformity; moreover, the *R*_a_ parameter of 2.4 nm indicates that the roughness remains very low. The surface potential map of the functionalized substrate (Fig. 3e) appears less uniform than the bare gold substrate. Banded regions with different potential contrast can be recognized together with smaller darker spots. These features in the potential map are most likely associated with the 10 μL deposition of 100 ng/μL List Capt-SH probe suspended in PBS 1X solution. The potential difference is only 16.8 mV between one dark spot indicated on the potential map and the surrounding region. Potential differences across the banded structure visible in the map are even lower (about 5 mV). Figure 3c, f shows topographic and Volta potential maps (30 μm × 30 μm) of the functionalized gold substrate after hybridization with the DNA of *L. monocytogenes.* The topographic map (Fig. 3c) displays several protruding features on the surface of the gold substrate which appears significantly rougher than the bare gold substrate (Fig. 3a) and the functionalized gold substrate (Fig. 3b). The *R*_a_ increases to 15.4 nm after the hybridization with *L. monocytogenes* DNA. The surface potential map in Fig. 3f reflects the heterogeneity of the topographic map. Several bright regions with high potential contrast are visible in the potential map. These bright regions appear significantly larger than the topographic features visible in Fig. 3a, clearly indicating that the potential contrast is not only generated by topographic features. This indicates that the hybridization process between the List Capt-SH probe and the DNA of *L. monocytogenes* leads to a marked modification of the surface potential. The potential difference is 65.8 mV between one of the bright features and the surrounding region (Fig. 3f).

### Textile OECT results

**Fig. 4:**
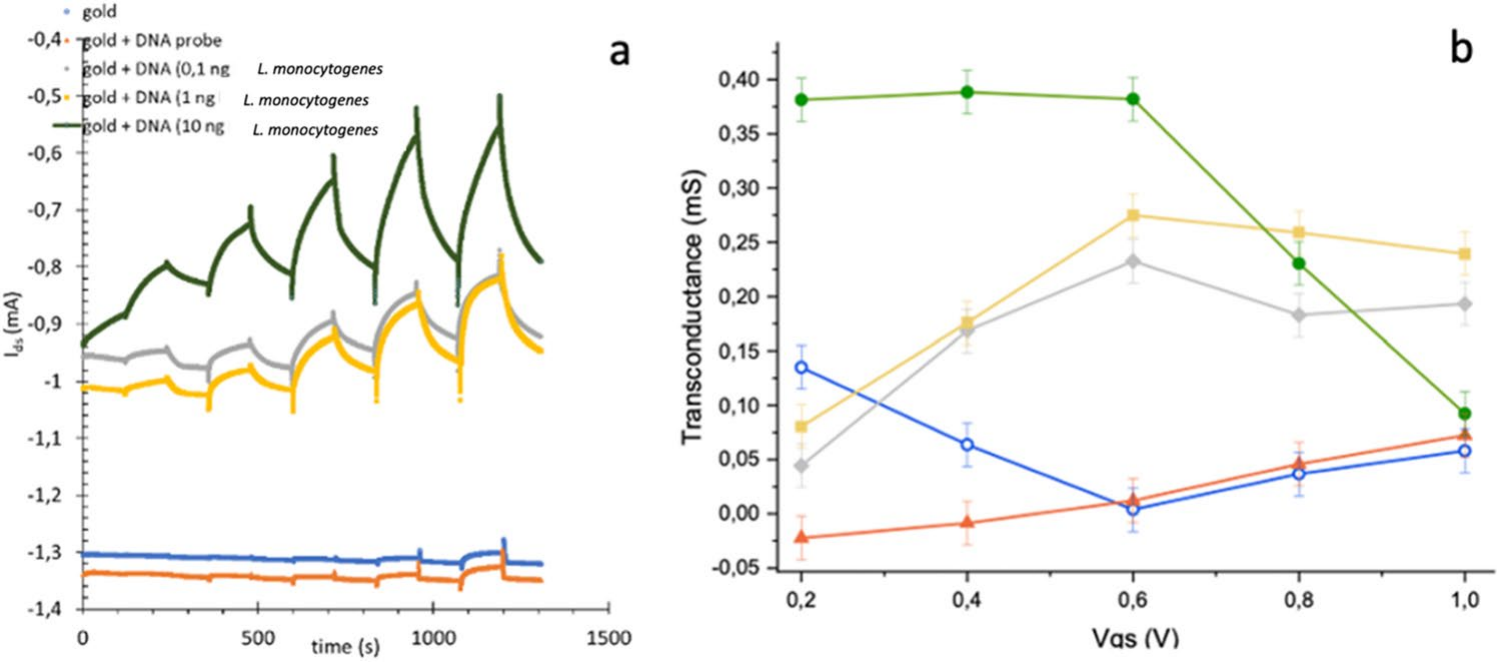
Modulations and characteristics of the textile OECT. **a** Channel current *I*_ds_ versus time for different gate voltage, ranging from 0.2 to 1 V with steps of 0.2 V alternate with 0 V, for different gate types in buffer solution (PBS 1X). **b** Transconductance, defined as the variation of channel current with respect to the variation of gate voltage (*g* = Δ*I*_ds_/Δ*V*_g_) values indicating great amplification. Bare gold substrate (blue line); gold substrate after functionalization with the List Capt-SH probe (red line); functionalized gold substrate after hybridization with 0.1 ng/μL DNA of *L. monocytogenes* ATCC 7644 (grey line); functionalized gold substrate after hybridization with 1 ng/μL DNA of *L. monocytogenes* ATCC 7644 (yellow line); functionalized gold substrate after hybridization with 10 ng/μL DNA of *L. monocytogenes* ATCC 7644 (green line)

To characterize the textile OECT biosensor, the channel current as a function of time at different gate voltages for a different amount of DNA of *L. monocytogenes* hybridized to the functionalized gold gate electrode was analyzed. *I*_ds_ versus time are reported in Fig. 4a.

Data obtained at 1 V for different concentrations of DNA of *L. monocytogenes* and eight samples are reported in Table 3. The response does not change significantly for the bare gold gate electrode values and the gold gate electrode functionalized with the List Capt-SH probe, whereas a significant modulation increase was observed after the hybridization of DNA of *L. monocytogenes* (from 0.1 ng to 10 ng) to the List Capt-SH probe immobilized onto the gate electrode. Furthermore, the gate current and transfer curve analysis confirmed this finding (Fig. S3).

**Table 3:** Sensor response (*R*) at 1 V gate voltage after deposition of different concentrations of DNA of *L. monocytogenes* ATCC 7644 (positive sample), *L. innocua* DSM 20649 (negative sample), and food samples SS1_p_, SS2_p_, SS1_4w_, SS2_4w_, RH1, RH2, CH1, and CH2 on the gold gate functionalized with List Capt-SH probe. Delta response (∆*R*) was calculated *R*-blank, the blank corresponds to the Au + probe, and standard deviation (SD) was reported for each measurement

Sample	Sensor response (*R*)	Delta response (∆*R*)	Standard deviation (SD)
Bare gold	0.0132	–	0.041
Au + probe 100 ng/μL	0.0176	–	0.003
*L. monocytogenes* ATCC 7644 DNA 0.1 ng/μL	0.1101	0.0925	0.005
*L. monocytogenes* ATCC 7644 DNA 1 ng/μL	0.1323	0.1147	0.001
*L. monocytogenes* ATCC 7644 DNA 10 ng/μL	0.3982	0.3806	0.008
*L. innocua* DSM 20649 DNA 100 ng/μL	0.1186	0.101	0.004
Experiment on food samples			
SS1_p_	0.1228	0.1052	0.002
SS2_p_	0.1215	0.1039	0.001
SS1_4w_	0.1236	0.106	0.002
SS2_4w_*	0.2244	0.2068	0.002
RH1	0.1229	0.1053	0.003
RH2*	0.1470	0.1294	0.004
CH1	0.1209	0.1033	0.001
CH2	0.1233	0.1057	0.002

^*^After 24 h incubation in enrichment broth

As explained by Tao et al. (2017), the sensing mechanism is based on the changes in surface potential due to the amount of DNA molecules on the gold gate electrode surface: since *C*_g_ and *C*_c_ are connected in series and *C*_g_ decreases following the DNA increasing on the gate, the potential drop at the gate/electrolyte interface. Therefore, the synergetic effect of gate capacitance with the surface potential of the gold gate electrode enlarges the increase in *V*_gs_, resulting in a modulation of the channel current (*I*_ds_) and then a sensor response increasing. The channel current (*I*_ds_) of the OECT is described by the equations (Bernards and Malliaras 2007; Bernards et al. 2008; Tao et al. 2017):

$$1: {\mathrm{PEDOT}}^{+}:{\mathrm{PSS}}^{-}+ {M}^{+}+ {e}^{-} \to {\mathrm{PEDOT}}^{0}+{M}^{+}:{\mathrm{PSS}}^{-}$$

$$2: R= \frac{\left|{I}_{\mathrm{ds}}- {I}_{\mathrm{ds}0}\right|}{{I}_{\mathrm{ds}0}}$$

$$3: {I}_{\mathrm{ds}}= \frac{q{\mu p}_{0}tW}{L{V}_{\mathrm{p}}}\left({V}_{\mathrm{p}}- {V}_{\mathrm{g}}^{\mathrm{eff}}+ \frac{{V}_{\mathrm{ds}}}{2}\right)\mathrm{ when }\left(\left|{V}_{\mathrm{ds}}\right|\ll \left|{V}_{\mathrm{p}}- {V}_{\mathrm{g}}^{\mathrm{eff}}\right|\right)$$

$$4: {V}_{\mathrm{p}}= \frac{q{p}_{0}t}{{C}_{\mathrm{i}}}$$

$$5: {C}_{\mathrm{i}}= \frac{{C}_{\mathrm{g}}{C}_{\mathrm{c}}}{\left({C}_{\mathrm{g}}+ {C}_{\mathrm{c}}\right) S}$$

$$6: {V}_{\mathrm{g}}^{\mathrm{eff}} = {V}_{\mathrm{g}}+ {V}_{\mathrm{offset}}$$

where *q* is the electronic charge, *μ* is the hole mobility, *p*_0_ is the initial hole density in the PEDOT:PSS before the *V*_gs_ application, *W* is the channel width, *L* is the channel length, *t* is the thickness of the PEDOT:PSS film, *V*_p_ is the pinch-off voltage, *V*^eff^_g_ is the applied effective gate voltage, *S* is the sensitivity (S), and *V*_offset_ is the offset voltage related to the potential drop at the two interfaces: gate/electrolyte and electrolyte/channel (Bernards et al. 2008; Cicoira et al. 2010; Tao et al. 2017).

Because both the immobilization of the DNA probe and DNA hybridization occurred on the gate electrode, the potential change (Δ*Ψ*) at the gate electrode surface is the only effect factor (Tao et al. 2017). Therefore,

$$7: {\Delta V}_{\mathrm{offset}}= \Delta \Psi$$

The surface dipole effected the surface potential change (Δ*Ψ*) as result of the intrinsic charge of the DNA (Tao et al. 2017):

$$8: \Delta \Psi = \frac{n{Q}_{\mathrm{DNA}}}{{\varepsilon }_{\mathrm{r}}{\varepsilon }_{0}} {t}_{\mathrm{DNA}}$$

where *n* is the density of DNA molecules on the surface, *Q*_DNA_ is the pure charge for one DNA molecule, *ε*_r_ is the relative dielectric constant of the DNA layer, *ε*_0_ is the dielectric permittivity of the free space, and *t*_DNA_ is the thickness of the DNA layer.

Table 3 shows the response of the textile OECT as function of the amount of DNA hybridized at the gold gate (*I*_ds_ current modulation (*R*)). To show the linearity of the system, data of sensor response reported in Table 3 were obtained with the same gate voltage (*V*_g_ = 1 V) for each gate type, to show the linearity of the system. High quantities of DNA on the gate resulted in high transconductance (*g*_m_) values which would indicate a greater amplification with a peak at *V*_g_ = 0.6 V (Fig. 4b); vice versa in complete absence of hybridized DNA or in the presence of the ssDNA probe alone, the amplification of the signal was lower.

The delta response (∆*R*) was calculated for each sample and represents the difference between the substrate after and before hybridization (*R*-blank). The blank corresponds to the Au + probe. The increase of the ∆*R* response with increasing of the DNA quantity was characterized by the linear equation $Y= 0.14414X+0.1959$ (*R*^2^ = 0.8074).

**Fig. 5:**
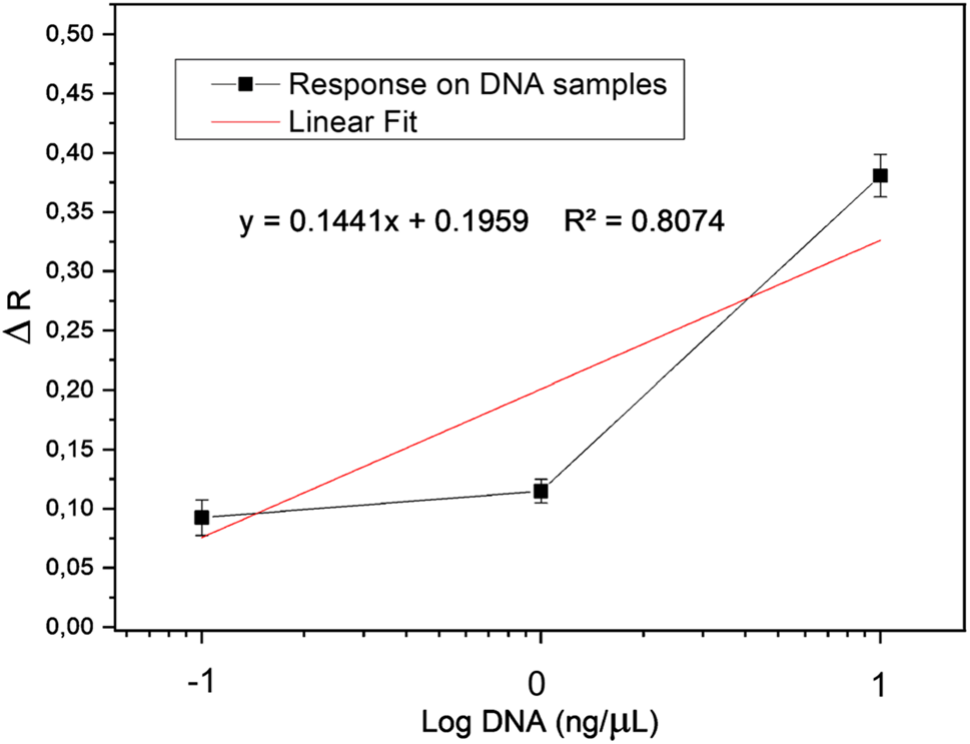
Response data in function of the Log DNA (ng/μL) of *L. monocytogenes* ATCC 7644 (*x*-axis) plotted towards the Δ*R* (*y*-axis)

The limit of detection (LOD) was determined based on the sensitivity and the average of the deviation standard (SD) according to the following equation:

$$\mathrm{LOD}=\left(k\times SD\right)/m$$

where *k* is the confidence level (*k* = 3), SD is the average SD of blank (Table 3), and *m* is the calibration sensitivity (the slope of the linear plot) (Harpaz et al. 2019) (Fig. 5). Therefore, the LOD of the system is 1.05 ng/μL, which correspond to 0.56 pM of *L. monocytogenes* ATCC 7644. This value is in agreement with LODs presented by other authors: Lin et al. (2011), Liang et al. (2019), and Peruzzi et al. (2021) reported a sensitivity of 1 nM, 10 pM, and 5 pM, respectively.

### Food sample analysis with OECT

The textile OECT biosensor was used on eight food samples analyzed by the *Listeria* Precis*™* method and PCR. The response obtained using DNA of *L. monocytogenes* ATCC 7644 at known concentrations shows increasing *R* values with the increase of the DNA concentration (Table 3).

To evaluate positive and negative samples, the delta response (∆*R* = *R*-blank) was considered. SS1_p_, SS2_p_, SS1_4w_, RH1, CH1, and CH2 samples showed the same values obtained from the negative sample *L. innocua* DSM 20649 (∆*R* = 0.101), while SS2_4w_ and RH2 samples (∆*R* values of 0.2068 and 0.1294, respectively) were considered positive for *L. monocytogenes.*

The values of the sensor response (*R*) of the probe alone and *L. innocua* DSM 20649 confirmed the probe selectivity which was already demonstrated by dot blot considering various microorganisms, including *L. innocua* DSM 20649 characterized by higher genomic similarity with *L. monocytogenes*.

The values of the delta response (∆*R*) of the food samples considered positive for *L. monocytogenes* ATCC 7644 are reported in Table 3.

## Discussion

The AFM 3D topographic maps obtained for the gold surfaces analyzed before and after the immobilization of the ssDNA probe confirmed that the functionalization of the surface was successful, indicating the good protocol used for the construction of the biosensor. In fact, the data related to the topographic and surface potential maps indicated a uniform functionalization process (Fig. 3), which produced a uniformly distributed DNA monolayer. This is a very important result considering that one critical step for the construction of an electrochemical genosensor is the immobilization of the ssDNA probe on the gold surface of the electrode (Xu et al. 2014).

The changes of the *I*_ds_ current absolute value (of the OECT biosensor) increased with the increase of the amount of *L. monocytogenes* DNA hybridized on the gate indicating the hybridization between the probe and the DNA target occurred as expected. As reported by Lin et al. (2011), the sensing mechanism is based on the changes in the surface potential due to the amount of DNA molecules on the gold gate electrode surface. According to Tao et al. (2017), since *C*_g_ and *C*_c_ are connected in series and *C*_g_ decreases following the DNA increasing on the gate, the potential drop at the gate/electrolyte interface and the channel/electrolyte interface, respectively, increases and decreases. Therefore, the synergetic effect of gate capacitance with the surface potential of the gold gate electrode enlarges the increase in *V*_g_, resulting in increased sensor response. Our textile OECT DNA sensor was able to detect specifically different amounts of DNA complementary to the DNA probe used through the sensing mechanism described. In detail, the increasing amount of target DNA hybridized on the gold gate electrode generated a potential variation at the interface of gate electrode/electrolyte solution, leading to the change of *I*_ds_ with a relative increase in *R*.

The textile structure in the channel presents different advantages (Gualandi et al. 2016), such as a high surface/volume ratio due to the microstructure of the textile with respect to a planar film of similar dimensions. Furthermore, the textile can absorb the fluid sample due to capillary forces, simplifying the sample uptake and increasing the possible application of the device in real environments (Coppedè et al. 2020).

Data from the *Listeria* Precis*™* method and PCR corresponded to results from our textile OECT biosensor: SS2_4w_ and RH2 samples were positive to *L. monocytogenes*, indicating the usefulness of the biosensor also for pathogen detection in a food complex matrix. This protocol allowed the detection of *L. monocytogenes* in a short time compared to the 5–6 days required by AFNOR or ISO methods and was not subjected to PCR inhibition by chemicals potentially present in the sample matrix (Schrader et al. 2012).

Moreover, data reported in Table 2 indicate the capability of the specific *L. monocytogenes* DNA probe to detect DNA target also in the presence of other contaminating bacteria. This is an important result in consideration of the reached limit of detection 1.05 ng/μL (corresponding to 0.56 pM of *L. monocytogenes* ATCC 7644), which can be further improved by optimizing the capture probe concentration on the gold gate. Considering our encouraging results, more samples will be tested to obtain validation of the proposed protocol specific for *L. monocytogenes* detection. Furthermore, new DNA probes specific for other relevant food pathogens will be designed and tested.

The proposed textile OECT biosensor is a very promising device for routine analyses of food samples considering its specificity towards *L. monocytogenes*, also in the presence of contaminating microorganisms as reported in Table 2. Data were also supported by the results obtained using *L. innocua* DSM 20649, bacteria characterized by a very high genome similarity*.* Due to its sensitivity, selectivity, and self-absorbing properties, the textile OECT shows different advantages with respect to other techniques.

It is useful for various applications, from neural interfaces (Khodagholy et al. 2015), printed circuits (Lee et al. 2017), clinical biomedical researches (Someya et al. 2016), and biological sensors (Nakatsuka et al. 2018); moreover, OECT biosensors can work on complex matrices (milk, blood) and can be coupled with various fabrication techniques (ElMahmoudy et al. 2017); it is simple to use, efficient, flexible, and integrable. It can be read with simple electrical measurements, and the signal is stable and reliable.

This system can be used for all food matrices that must guarantee the absence of the pathogen in 25 g of sample and instead of analysis methods with an enrichment step required for foods with zero tolerance towards *L. monocytogenes.* A rapid pathogen detection can help in reducing economic losses due to both food recalls and hospitalization. Nevertheless, the developed method can be improved increasing the sensitivity to match the EU microbiological criterion for *L. monocytogenes* which is set as ≤ 100 CFU/g for food products on the market.

## Supplementary Information

Below is the link to the electronic supplementary material.

Supplementary file1 (PDF 206 KB)

## Data Availability

All data generated or analyzed during this study are included in this published article (and its supplementary information files).
