# Supplementary material for: Detection of Listeria monocytogenes in foods with a textile organic electrochemical transistor biosensor
Source: Appl Microbiol Biotechnol. 2023 May 5;107(11):3789–800. doi: 10.1007/s00253-023-12543-y (PMC10175343; doi:10.1007/s00253-023-12543-y)
Supplement: Supplementary file 1 — Supplementary file1 (PDF 206 KB) [file 253_2023_12543_MOESM1_ESM.pdf]

SUPPLEMENTARY INFORMATION

Manuscript:

Detection of *Listeria monocytogenes* in foods with a textile organic electrochemical transistor biosensor

Journal: Applied Microbiology and Biotechnology

Authors:
Priya Vizzini[1], Elena Beltrame[1], Nicola Coppedè[2], Filippo Vurro[2], Francesco Andreatta[3], Emanuela Torelli[4], Marisa Manzano[1*]

[1]Department of Agriculture Food Environmental and Animal Sciences, University of Udine, 33100 Udine, Italy
[2] Institute of Materials for Electronics and Magnetism IMEM CNR Parco Area delle Scienze, 43124 Parma, Italy
[3] Polytechnic Department of Engineering and Architecture, University of Udine, 33100 Udine, Italy
[4] Interdisciplinary Computing and Complex BioSystems (ICOS), Centre for Synthetic Biology and Bioeconomy (CSBB), Devonshire Building, Newcastle University, Newcastle upon Tyne, NE1 7RX, United Kingdom.

Corresponding author:
Marisa Manzano; marisa.manzano@uniud.it; Dipartimento di Scienze AgroAlimentari, Ambientali e Animali, via Sondrio 2/A, 33100 Udine, Italia
phone: +390432558127
fax: +390432558130

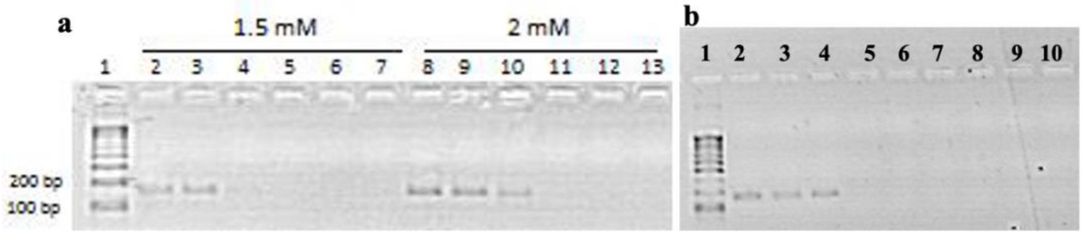

Fig. S1 a) $MgCl_2$ optimization at 48°C annealing. Line 1, 100 bp DNA ladder (Promega); Lines 2-7, $MgCl_2$ 1.5 mM; lines 8-13, $MgCl_2$ 2 mM; Lines 2, 8, *L. monocytogenes* 1/2c ATCC 7644; lines 3, 9, *L. monocytogenes* 1/2a DSM 112143; lines 4, 10, *L. monocytogenes* 1/2bDSM 19094; lines 5, 11, *L. innocua* DSM 20649; lines 6, 12, *L. ivanovii* DSM 12491; lines 7, 13 blank.

b) Amplicons obtained using DNAs extracted from reference strains and salmon and ham samples. Line 1:100 bp Ladder (100 bp, Sigma), line 2: Sliced salmon sample $SS5_{4w}$; line 3: Raw ham sample RH2; line 4: *Listeria monocytogenes* ATCC 7644; line 5: $SS5_p$; line 6: Sliced salmon $SS6_{4w}$; line 7: Raw ham sample RH4; line 8: Raw ham sample CH2 line 9: *L. innocua* DSM 20649; line 10: blank

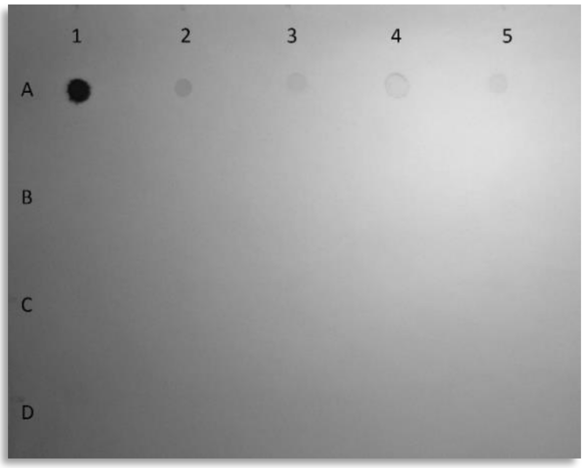

Fig. S2 Dot blot results of List Capt probe at 200 ng/µL using positive and negative controls at 100 ng/µl, hybridization temperature 40°C. Row A, A1: sequence complementary to the probe (positive control); A2: *Listeria monocytogenes* 1/2c ATCC 7644; A3: *L. monocytogenes* 1/2a DSM 112143; A4: *L. monocytogenes* 1/2b DSM19094; A5: *L. monocytogenes* 4b DSM 15675. Row B, B1: *L. innocua* DSM 20649; B2: *L. ivanovii* DSM 12491; B3: *S. enterica* DSM 9145; B4: *E. coli* DSM 1103. Row C, C1: *Bacillus cereus* DSM 2301; C2: *Campylobcter jejuni* DSM 49943; C3: *Lactoplantibacillus plantarum* ATCC BAA793; C4: *Lacticaseibacillus paracasei* DSM 5622; C5: *Lacticaseibacillus rhamnosus* ATCC 53103; D1: *Levilactibacillus brevis* DSM 20054

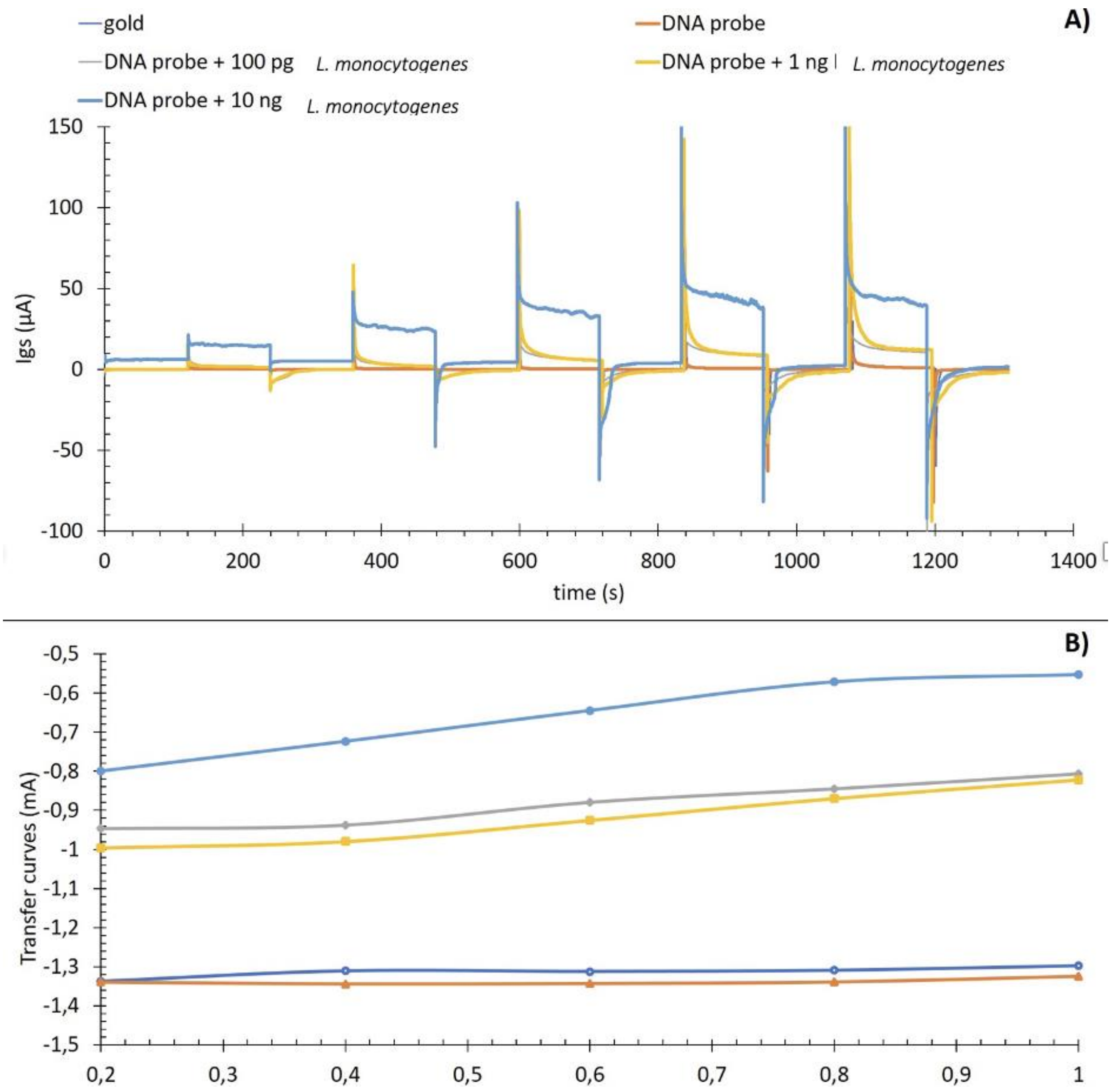

Fig. S3 Gate current and transfer curves of OECT composed by different gate electrode types. A) Gate current I_{gs} versus time for different gate voltage, ranging from 0.2 to 1 V with steps of 0.2 V alternate with 0 V, for different gate types in buffer solution (PBS 1X); B) Transfer curves as a function of gate voltage from 0.2 to 1 V with 0.2 V step for different gate types in buffer solution (PBS 1X). Bare gold substrate (blue line); gold substrate after functionalization with the List Capt-SH probe (red line); functionalized gold substrate after hybridization with 0.1 ng/ µL DNA of *L. monocytogenes* (gray line); functionalized gold substrate after hybridization with 1 ng/ µL DNA of *L. monocytogenes* (yellow line); functionalized gold substrate after hybridization with 10 ng/ µL DNA of *L. monocytogenes* (light blue line)
